# ﻿Revision of the North American genus *Supralathosea* Barnes & Benjamin (Lepidoptera, Noctuidae, Oncocnemidinae) with description of two genera and three species

**DOI:** 10.3897/zookeys.1228.141682

**Published:** 2025-02-21

**Authors:** Lars G. Crabo, Kevin Keegan

**Affiliations:** 1 Adjunct Faculty, Department of Entomology, Washington State University, Pullman, Washington, USA Washington State University Pullman United States of America; 2 Carnegie Museum of Natural History, Pittsburgh, Pennsylvania, USA Carnegie Museum of Natural History Pittsburgh United States of America

**Keywords:** Amphipyrinae, Chihuahuan desert, DNA barcode, key, Madrean Archipelago, Oncocnemidinae, owlet moths

## Abstract

The noctuid genus *Supralathosea* Barnes & Benjamin (Noctuidae, Oncocnemidinae) is revised to include three species for the United States of America, *Supralathoseababoquivariensis* Barnes & Benjamin from southeast Arizona, *Supralathoseayavapai***sp. nov.** from central Arizona, and *Supralathoseasolastella***sp. nov**. from Texas. Two genera are described for species formerly included in *Supralathosea*. *Infralathosea***gen. nov.** includes *Infralathoseapronuba***comb. nov.** from Arizona and New Mexico and *Infralathoseaunicornis***sp. nov.** from west Texas. *Eulathosea***gen. nov.** contains only *Eulathoseaobtusa* Smith, **comb. nov.** from Arizona. A key to genera and species is presented and adults and genitalia of all taxa are illustrated. *Infralathosea* and *Eulathosea* are assigned to Oncocnemidinae based on molecular evidence.

## ﻿Introduction

*Supralathosea* Barnes & Benjamin, 1924 (Lepidoptera, Noctuidae) species are relatively nondescript medium-sized noctuid moths that are known from Arizona, New Mexico, and Texas in the United States but undoubtedly also occur in Mexico. The males of all species have bipectinate antennae and forewings with a nearly uniform pale to dark gray ground color, usually with faint noctuiform patterning modified with a black line along the trailing margin, and white or off-white hindwings. Females are darker gray, with more pronounced forewing patterning and gray suffusion on the hindwings. *Supralathosea* are uncommon in collections despite flying during much of the year, including in wintertime.

*Supralathosea* was described for *Supralathoseababoquivariensis* Barnes & Benjamin, 1924 (Barnes and Benjamin 1924). The genus remained monotypic until 1989 when *Catabenapronuba* Barnes & McDunnough, 1916 and *Cuculliaobtusa* Smith, 1909 were reassigned to *Supralathosea* from the genera in which they were originally described in the non-annotated Lepidopterorum Catalogus series (Poole 1989). *Supralathosea* has not been formally revised, and these three species were retained in the genus in subsequent North American check lists (Lafontaine and Schmidt 2010; Pohl and Nanz 2023). Although they share superficial similarities, these species are structurally distinct, differing in the morphology of the male antennae, male genital capsules and vesicae, and female bursae and egg laying apparatuses. Here we describe two new genera, *Infralathosea* gen. nov. and *Eulathosea* gen. nov., to provide a more cogent arrangement of these taxa. In addition, we describe two new species of *Supralathosea* and one new species of *Infralathosea*, all from southwestern United States.

Barnes and Benjamin (1924) placed *Supralathosea* in Cuculliinae (sensu Hampson 1903) where it was retained by McDunnough (1938) and Hodges et al. (1983). Cuculliinae is now defined more narrowly (Poole 1995), and most genera included in it historically have been dispersed to a number of different subfamilies (Lafontaine and Schmidt 2010; Pohl and Nanz 2023). *Supralathosea* was reassigned from Cuculliinae to Psaphidinae by Troubridge (2008), and shortly thereafter to Amphipyrinae, Psaphidini, Feraliina by Lafontaine and Schmidt (2010). Keegan et al. (2019, 2021) subsequently showed Amphipyrinae to be polyphyletic based on an analysis of nuclear and mitochondrial DNA sequences, and they reassigned *Supralathosea* to Oncocnemidinae as part of a massive restructuring of Amphipyrinae and related taxa.

## ﻿Materials and methods

Wing pattern and genitalia structure terminology follow Lafontaine (2004). Deep lateral invaginations forming a transverse channel across abdominal segment A1 dorsal to the tympanum, typically found in many oncocnemidine genera (Troubridge 2008; Keegan et al. 2021), is herein called the “A1 transverse channel.” A spike-like extension from the ventral cucullus in *Supralathosea* is the pollex. Forewing lengths were measured from base to apex to the nearest half millimeter, excluding the fringe. All measurements and ratios in the genus and species descriptions and Diagnosis sections are based on the examined material, and are approximations. For readability we have omitted a “~” as an indicator of uncertainty prior to any numerical values. It is likely that individual specimens will be found that vary slightly from the descriptions.

Genitalia were prepared using standard methods (Hardwick 1950, Lafontaine 2004). Abdomens were macerated in hot 10% potassium hydroxide for 25–30 minutes. Initial dissections were performed in water followed by hardening in 95% isopropyl alcohol. Male vesicae and female bursae were inflated. Preparations were stained with orcein (Sigma Chemical Company, St. Louis, Missouri) and mounted in Euparal (Bioquip Products Inc., formerly of Rancho Dominguez, California) on glass slides with elevated cover glass to preserve the three-dimensional configuration of the structures. Some illustrated genitalia preparations from NMNH are stained blue, likely using Chlorazol black. Unless specified otherwise, female genitalia comparisons to “A8” refer to the anteroposterior length of segment A8.

The 658 base pair DNA “barcode region” of the mitochondrial cytochrome *c* oxidase subunit 1 (CO1) (“barcode”) was used to assess intra- and interspecific molecular variation within each genus. Legs from dried specimens were submitted to the Barcodes of Life Data System (BOLD) at the University of Guelph (Ontario, Canada) where they were subjected to standard DNA extraction, amplification, and sequencing protocols (Hebert et al. 2003) or sequenced by KK as described in Keegan et al. (2019). Barcodes were compared to pre-existing material at BOLD using the Kimura-2-Parameter distance model as implemented on http://www.barcodinglife.org.

Intergeneric phylogenetic relationships were evaluated by inferring a phylogeny in RAxML v. 8.2.12 (Stamatakis et al. 2008) with 1000 non-parametric bootstraps (BS) on CIPRES (Miller et al. 2010) based on previously available and newly generated sequences of five nuclear genes and mitochondrial CO1 as described in Keegan et al. (2019). We considered clades with BS ≥ 70 to be well supported (Hillis and Bull 1993; Lemoine et al. 2018).

Distribution data includes coordinates associated with photographs of live moths on the websites iNaturalist (iNaturalist community 2024) and BugGuide (VanDyk et al. 2024), vetted by LGC. Distribution data obtained from internet photographs are depicted as squares to distinguish them from data from examined specimens (circles) on the maps (Figs 33, 34).

### ﻿Repository abbreviations

**AMNH**American Museum of Natural History, New York, New York, USA

**CMNH**Carnegie Museum of Natural History, Pittsburgh, Pennsylvania, USA

**CNC**Canadian National Collection of Insects, Arachnids, and Nematodes, Ottawa, Ontario, Canada

**DLW** Dave Wikle personal research collection, Whittier, California, USA

**JV** James Vargo personal research collection, Mishawaka, Indiana, USA

**LACM**Natural History Museum of Los Angeles County, Los Angeles, California, USA

**LGC** Lars Crabo personal research collection, Bellingham, Washington, USA

**NMNH**National Museum of Natural History, Washington, D. C., USA

## ﻿Results

Our molecular dataset consisted of at most six genes and 5539 base pairs for 23 taxa. Represented among these taxa were three non-Oncocnemidinae outgroups representing the noctuid subfamilies Plusiinae, Acontiinae, and Cuculliinae. The genera *Eulathosea*, *Infralathosea*, and *Supralathosea* formed a well-supported group (BS = 91) (Fig. 35). We found *Supralathosea* to be monophyletic (BS = 100) with *Eulathosea* sister to it (BS = 85), and we recovered *Infralathosea* as a clade (BS = 69) sister to other two genera. Within *Supralathosea*, we found *S.baboquivariensis* sister to the remaining taxa in the genus that formed a well-supported clade (BS = 83). The relationship between these remaining *Supralathosea* taxa (*Supralathoseayavapai* sp. nov. and *Supralathoseasolastella* sp. nov.) is unresolved in our tree.

### ﻿Key to adults of *Supralathosea* sensu Pohl and Nanz (2023) for North America north of Mexico

**Table:** 

1	Hindwing two toned, white with broad uniform blackish gray marginal band; forewing gray with orange-tan scales in cell near reniform spot (Figs 17, 18)	***Eulathoseaobtusa***
–	Hindwing white, off white, or gray, at most with a weak marginal band of powdery gray scales intermixed with the ground color; forewing gray, lacking warm colors (Figs 1–16)	**2**
2	Male antenna bipectinate; anterior female ductus bursae > 1.5 × width of A8, thickened, crenulate (Figs 26–29); FW transverse lines either obsolete or normal, extending completely across wing when visible (Figs 1–12)	**3 (*Supralathosea*)**
–	Male antenna beaded; anterior female ductus bursae tubular, < 1 × width of A8 (Figs 30, 31); FW antemedial and postmedial line segments fused to form arcs from anterior and posterior margins (Figs 13–16)	**5 (*Infralathosea*)**
3	Pollex of male valve triangular, length 2 × base width (Fig. 22); thick anterior segment of female ductus bursae wider than anteroposterior length of corpus bursae (Fig. 29); central Arizona	***Supralathoseayavapai***
–	Male pollex rod-like, slender from base to apex (Figs 19–21); anterior female ductus bursae width less than anteroposterior length of corpus bursae (Figs 26–28); southern Arizona and Texas	**4**
4	Male forewing grainy charcoal gray with dark veins, darker gray scales in subterminal area and on trailing margin in some individuals (Figs 1, 3); female hindwing with uniform dark marginal shade (Fig. 2); Madrean Archipelago of southeast Arizona	***Supralathoseababoquivariensis***
–	Male forewing smooth medium to pale gray with black trailing margin or distinct black transverse lines (Figs 4, 5, 7); female hindwing marginal area variable, white or with patchy gray suffusion (Figs 6, 8, 9); Texas	***Supralathoseasolastella***
5	Male vesica without apical stout cornutus (Fig. 23b); female hindwing gray (Fig. 14); Arizona and New Mexico	***Infralathoseapronuba***
–	Male vesica with apical stout cornutus (Fig. 24b); female hindwing white (Fig. 16); west Texas	***Infralathoseaunicornis***

#### ﻿Genus Supralathosea

Taxon classificationAnimaliaLepidopteraNoctuidae

Barnes & Benjamin, 1924

18DA78D8-D6FB-516C-8B10-6081BD302498

##### Type species.

*Supralathoseababoquivariensis* Barnes & Benjamin, 1924; by monotypy.

##### Diagnosis.

*Supralathosea* species are pale to dark gray moths with forewings of 12.0–18.5 mm in length, adorned with very narrow scales. Males can usually be recognized by the combination of broad bipectinate antenna, relatively unmarked forewing with black posterior margin, and white hindwing with black thin incomplete terminal line and veins. Females are less distinctive, with filiform antenna, mottled darker gray forewing with more distinct transverse lines, pale-filled orbicular and reniform spots lacking dark outlines, and white hindwing often heavily dusted with gray. Although similar, males of the *Supralathosea* species can be identified by their superficial appearance. The females are more similar and are best identified by association with the males or by locality.

Male genitalia have an uncus that is bent nearly 90° at the base. The valve is bluntly pointed near the apex, has a spike-like pollex but no corona, and the clasper ampulla is cow-horn-shaped. The vesica of the phallus is bulbous with multiple spike-like setae, resembling a medieval mace.

Females have a unique broad, thick, crenulate anterior segment of the ductus bursae abutting the corpus bursae, somewhat like beet or walnut half. The corpus bursae is asymmetric, pear-shaped with narrower right side directed slightly posteriad, and lacks signa.

Below we provide a redescription for *Supralathosea* in light of the recognition of *Eulathosea* and *Infralathosea*, and descriptions for the new species of *Supralathosea*.

##### Redescription.

Adults medium size (FW length 12.0–18.5 mm) with pale to dark gray forewings, usually with very faint noctuiform patterning (lines distinct in south Texas populations) and black posterior margin. ***Head*** – Male antenna broadly bipectinate, rami 2.5–3.0 × central shaft width, covered densely except on dorsum with short cilia; female antenna filiform. Eye lacking interommatidial setae, strongly lashed posteriorly. Frons bulging slightly; dorsal head scales projecting slightly over frons with small paramedian tufts. Haustellum present. Labial palpus with short strap-like and long ventral and lateral black hair-like scales. ***Thorax*** – Collar with median crest, prominent in resting live moths, thin, white-edged black line across base; tegulum and dorsum gray, darkest dorsally. ***Wings***: Forewing elongate with slightly pointed apex in males, slightly wider and rounder in females. Scales long, narrow, finely serrate, gray, translucent white, and gray-edged or gray-tipped white; ground color uniform pale gray to charcoal gray, posterior margin usually dark gray or black; subterminal area with three indistinct dark patches. Lines variable, very faint or limited to indistinct oblique dark marks on costa and vestiges of the postmedial line on the veins in males; those of some male *Supralathoseasolastella* and all females are dark gray to black; basal line absent or a dark spot on costa; antemedial line double, inner component weak, pale filled, sinuous, convex with apex in fold; postmedial line similar, outer component weak, sinuous or dentate, prominently convex around cell with notch opposite reniform, segment posterior to Cu drawn basad in fold then perpendicular to posterior margin; medial line dark gray, nearly parallel to postmedial line; subterminal line pale, indistinct; terminal line of intervenal dark spots. Orbicular and reniform spots pale with dark centers, small and inconspicuous; claviform spot absent. Hindwing scales small translucent and long hair-like, pure white or off white, darker gray scales on portions of veins and terminal line in males; similar white or pale gray with discal spot, medial line, and distal gray suffusion in females. ***Legs***: Scales mostly dark gray, scattered white; lacking tibial claw or other modifications; tarsal segments with three rows of setae, dark gray with distal white rings. ***Abdomen*** – Lacking A1 transverse channel, brush organs, and coremata. Scales pale to brownish gray; weak darker dorsal tufts on proximal segments. ***Male genitalia***: Uncus relatively short, directed posteriad at base then bent 90° ventrad, cylindrical with small apical hook. Juxta broad, shield shaped with variable length ventral extension. Valve rhomboid, length 2.8–3.8 × width, distal 1/3 broadest, tapered to a subapical point with spike-like pollex at anal margin, lacking expanded cucullus or corona. Sacculus strong, extending to mid-valve, convex to near costa. Clasper base a bar near ventral margin distal to sacculus, ampulla arising at valve distal 1/3, cow-horn-shaped with > 90° curve dorsad and basad. Phallus length 3.5–4 × width, dorsal apex with sclerotized crenulate band with minute spikes. Vesica with 90° subbasal and mesial bends, apex posterior to proximal phallus, mesial segment ballooned posterior and leftward, covered by 50–70 spike-like cornuti, longest 6–7 at dorsal margin; subapical vesica with smaller patch of shorter thinner cornuti on convex segment or small diverticulum. ***Female genitalia***: A7 tergite thick, large, lateral margins converging at ventral midline, covering small weakly sclerotized sternite; A7 pleural membranes leathery, redundant, covered densely with dark scales. Segment A8 width 1.4–1.9 × length. Papilla analis 1 × A8, mesiolaterally compressed, apex blunt, covered sparsely with uniform hair-like setae; not protruding in situ. Posterior apophysis 1.9–2.8 × A8; anterior apophysis 0.6–0.8 × posterior apophysis. Ostium bursae U-shaped, leathery with crenulate margin and lightly sclerotized broad V-shaped dorsal and ventral postvaginal plates. Ductus bursae with distinct anterior and posterior segments: shorter posterior ductus segment membranous, conical, broadest at ostium; longer anterior segment irregular, thick, rubbery, bulb-like with broad base at transverse or slightly angled junction to corpus bursae, length 1.7–2.2 × A8, width 1.0–1.2 × length, internally amorphous, seemingly lacking a lumen. Corpus bursae membranous, lacking signa, length 2–2.5 × A8, width 1.2–1.5 × length, pear shaped, apex right posterior with ductus seminalis at tip.

##### Higher classification.

*Supralathosea* is assigned to Oncocnemidinae (Keegan et al. 2021).

##### Distribution and biology.

This genus occurs in southwestern United States, where it has been found in Arizona and Texas. Several of the species undoubtedly occur in Mexico. Adults of most *Supralathosea* species fly throughout much of the year, but many records are from winter, December through February. Females are found much less commonly than males. The larva of *S.solastella* feeds on ash (*Fraxinus* sp., Oleaceae) (D. Wagner, pers. comm.). The early stages of the other species are unknown.

##### Discussion.

United States populations of *Supralathosea* are distributed in four seemingly allopatric populations, three of which abut the border with Mexico. We have no information about the distribution of the genus in northern Mexico, but it seems certain that at least one or two *Supralathosea* species also occur there based on their known distributions in the United States. With this in mind, we arrange the United States populations into three species. Two relatively uniform and superficially distinctive species from separate parts of Arizona are supported by several characters, including distinct DNA sequences (*S.baboquivariensis*) or male genitalia (*Supralathoseayavapai*). The other two populations from Texas are treated as a third species (*Supralathoseasolastella*). While these Texas populations are relatively distinct superficially, there are no significant genitalic or barcode differences between them to indicate that they are separate species. We suspect that the superficial differences represent clinal variation, but this can be neither confirmed or refuted until more is known about *Supralathosea* in northern Mexico.

*Supralathoseababoquivariensis* is relatively distinct genetically, with barcodes differing from those of other *Supralathosea* species by almost 4% and strong support in our RAxML maximum-likelihood analysis (Fig. 35). The other two species have several barcode haplotypes differing by less than 1.5%, and are unresolved in our multi-gene analysis. This is not unusual, as distinct species of other noctuid genera have similar or identical barcodes, including many species of *Euxoa* Hübner, *Abagrotis* Smith, and *Lasionycta* Aurivillius (Zahiri et al. 2014).

*Supralathosea* species lack a dorsal A1 transverse channel, an anatomic feature common to many Oncocnemidinae genera. Troubridge (2008) considered this to be the defining character of the subfamily, although he noted that it is also occurs in some diurnal species of Agaristinae (e.g., *Alypia* Hübner) and Stiriinae (e.g., *Annaphila* Grote). He moved several genera that lack this character to various non-oncocnemidine subfamilies, including assigning *Supralathosea* to Psaphidinae (now Psaphidini). Based on the DNA-based studies of Keegan et al. (2019, 2021) it is now clear that not all genera in the subfamily express this character, including *Supralathosea*, *Infralathosea*, *Eulathosea*, and at least some species of *Leucocnemis* Hampson.

#### ﻿ Supralathosea baboquivariensis

Taxon classificationAnimaliaLepidopteraNoctuidae

Barnes & Benjamin, 2024

4AD6F365-B44E-5A1F-9163-600C1952598B

**Figures 1–18.**
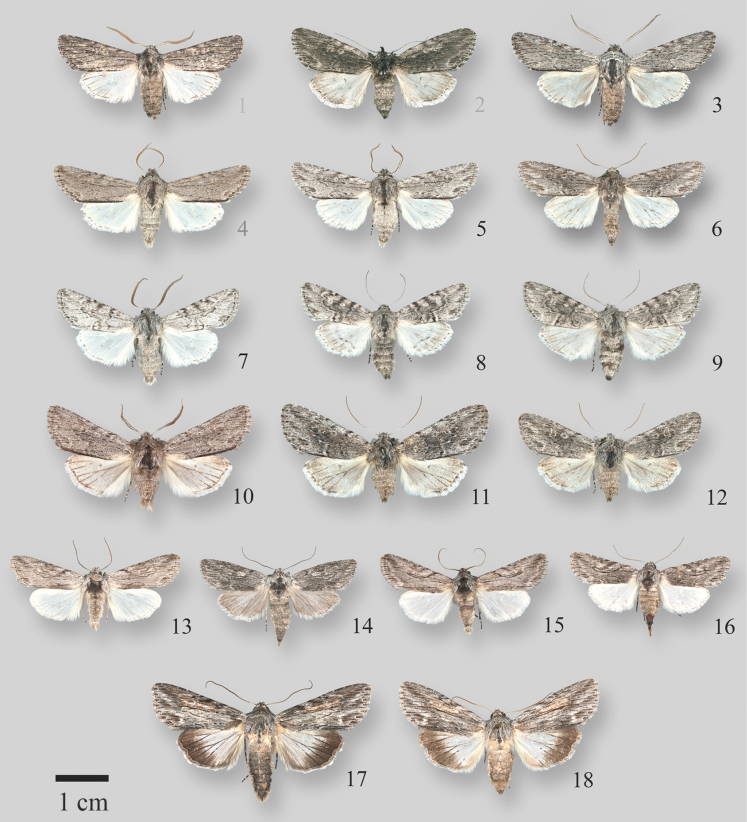
*Supralathosea*, *Infralathosea*, and *Eulathosea* adults **1***Supralathoseababoquivariensis* male, USA, Arizona, Pima County **2***S.baboquivariensis* female, USA, Arizona, Pima County **3***S.baboquivariensis* male, USA, Arizona, Cochise County **4***Supralathoseasolastella* HT male, USA, Texas, Brewster County **5***S.solastella* male, USA, Texas, Brewster County **6***S.solastella* female, USA, Texas, Brewster County **7***Supralathoseasolastella* male, USA, Texas, Zapata County **8***S.solastella* female, USA, Texas, Zapata County **9***S.solastella*, USA, Texas, Zapata County **10***Supralathoseayavapai* USA, Arizona, Yavapai County **11***S.yavapai* USA, Arizona, Yavapai County **12***S.yavapai* USA, Arizona, Yavapai County **13***Infralathoseapronuba* male, USA, Arizona, Yavapai County **14***I.pronuba* female, USA, New Mexico, Lincoln County, **15***Infralathoseaunicornis*, male, USA, Texas, Brewster County **16***I.unicornis*, female, USA, Texas, Brewster County **17***Eulathoseaobtusa* male, USA, Arizona, Cochise County **18***Eulathoseaobtusa* female, USA, Arizona, Yavapai County.

**Figures 19–22.**
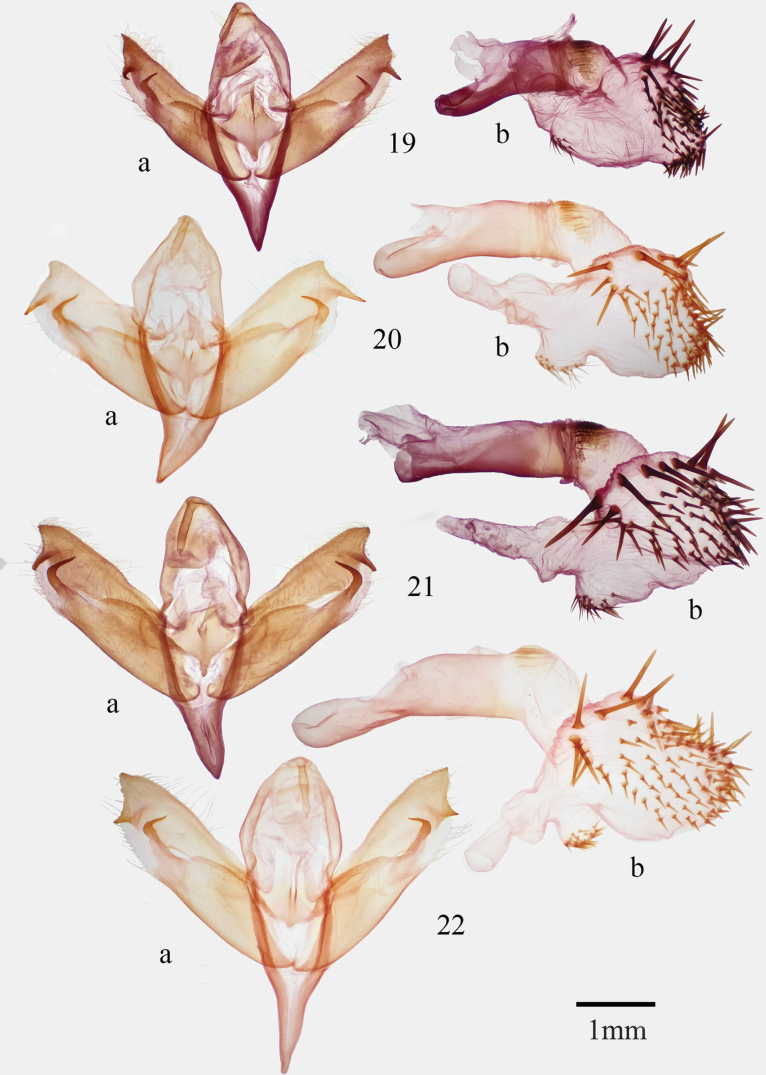
*Supralathosea* male genitalia **19***Supralathoseababoquivariensis***20***S.solastella* (Brewster County, Texas) **21***S.solastella* (Zapata County, Texas) **22***S.yavapai*.

Figs 1–3 , 19 , 26 , 33 (Map)

Supralathosea baboquivariensis Barnes & Benjamin, 1924: 133.

##### Type material.

Holotype male. USA, Arizona, Pima County, Baboquivari Mountains. NMNH [examined].

##### Diagnosis.

*Supralathoseababoquivariensis* can be distinguished from the only other *Supralathosea* species in Arizona, *S.yavapai*, by its slightly smaller size (FW length of *S.baboquivariensis* < 15.5 mm; *S.yavapai* > 16.5 mm) and its pure white rather than off white hindwing ground color. The male pollex is thin and spike-like in *S.baboquivariensis*, shorter and broadly triangular in *S.yavapai*. The female bursa of *S.baboquivariensis* is rounder than that of *S.yavapai*.

*Supralathoseababoquivariensis* is similar in size to *S.solastella*, found in Texas. The forewing of male *S.baboquivariensis* is dark charcoal gray with a rough texture; that of *S.solastella* is smoother, either medium gray with thick black posterior margin (west Texas) or pale gray with distinct noctuiform pattern of transverse lines (south Texas). The hindwing ground color of both species is white, but the veins of *S.baboquivariensis* are nearly completely dark gray, whereas those of *S.solastella* are white or have less extensive gray scales limited to the positions of the postmedial and terminal lines. Females of *S.solastella* are more similar to *S.baboquivariensis* than the males, but tend to be paler gray with paler hindwings, especially in south Texas. The male valve and vesica and female bursa copulatrix of *S.baboquivariensis* are less massive than those *S.solastella*. This is evident in the figures, but the differences are subtle and difficult to appreciate without a side-by-side comparison.

Barcodes of *S.baboquivariensis* (BOLD:ADP1765) differ from those of other *Supralathosea* species by approximately 4%.

##### Distribution and biology.

*Supralathoseababoquivariensis* is uncommon in collections. It occurs in the mountains of southeastern Arizona, with almost all examined specimens from the Baboquivari Mountains. A single male (Fig. 3) from Ash Canyon on the east slope of the Huachuca Mountains (NMNH) is slightly smoother dark gray than topotypical specimens (Fig. 1). It is assigned to *S.baboquivariensis* until more material is available for examination. Adults fly throughout the year, mostly during summer and winter. Examined specimens were collected during June, July, October, and December. The early stages are unknown.

**Figures 23–25.**
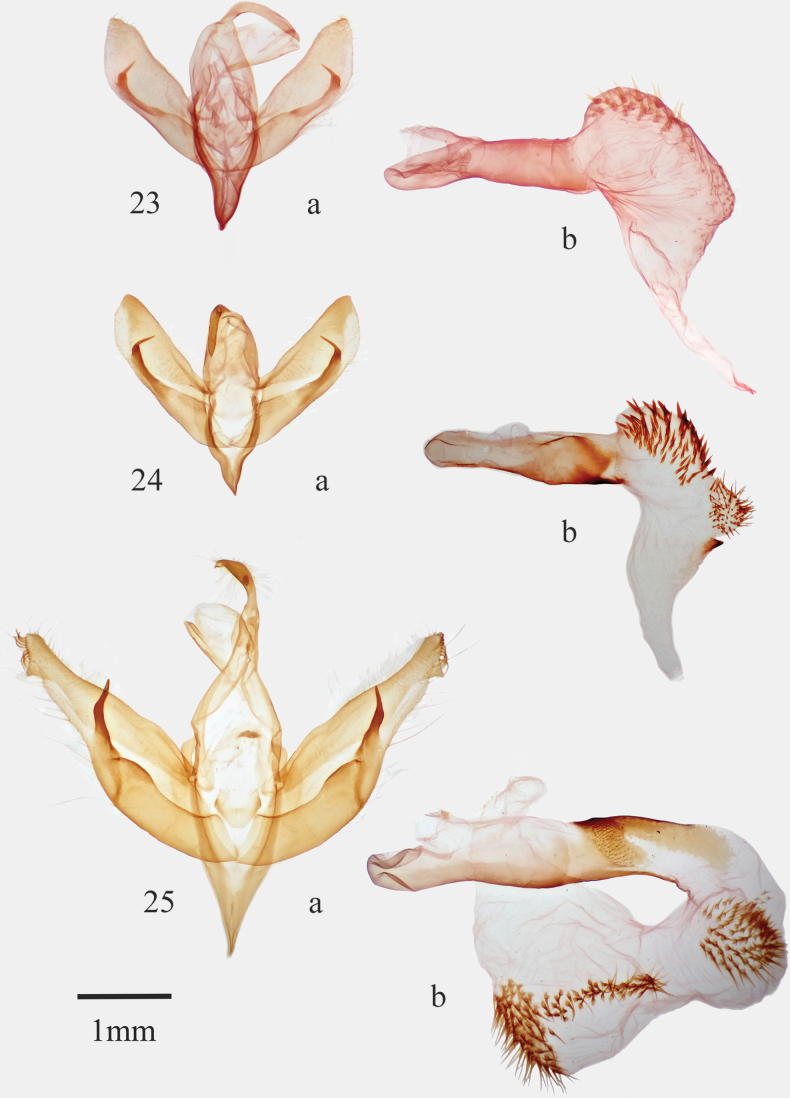
*Infralathosea* and *Eulathosea* male genitalia **23***Infralathoseapronuba***24***I.unicornis***25***Eulathoseaobtusa*.

#### ﻿ Supralathosea solastella sp. nov.

Taxon classificationAnimaliaLepidopteraNoctuidae

BD5DDA7D-2C24-5410-A863-B9C8B89F50D7

https://zoobank.org/25A24221-E5C1-4B18-8900-32B39F849B3C

Figs 4–9 , 20 , 21 , 27 , 28 , 33 (Map)

##### Type material.

***Holotype*, male.** [USA]: Texas: [Brewster County]: Big Bend Nat[ional] Park, Gov[ernment] Spring, 31 III [19]65, A. & M. E. Blanchard. NMNH. ***Paratypes*.** 26 males, 5 females. **USA: Texas**: Brewster County: Big Bend Nat[ional] Park, Basin, A. & M. E. Blanchard, 8 VII [19]64 (1 male); 10 VII [19]64 (1 male); 10 VII [19]64 / Genitalia slide by RWP [female] USNM 45184 (1 female); 11 VII [19]64 (3 males, 2 females); 11 VII [19]64 / Barcode of Life, DNA voucher specimen CCDB-20821-C12, BOLD Proc. ID LNAU55033-13 / USNMENT 00906843 (1 male); 29 III [19]65 (1 male); 1 VII [19]65 (1 male, 1 female); 27 IX [19]65 (1 male); 2 X [19]66 (1 male); 9 IV 1967 (1 male); Big Bend Nat. Park, The Basin, 9 V 1959, M. R. MacKay / Genitalia CNC slide # 13940 male (1 male); Big Bend Nat[ional] P[ar]k, Chisos Basin, 30 V [19]81, Leg. E. C. Knudson / Barcode of Life, DNA voucher specimen CCDB-20821-C08, BOLD Proc. ID LNAUS 5029-13/USNMENT 00906839 (1 male); Big Bend Nat[ional] Park, Gov[ernment] Spring, A. & M. E. Blanchard, 31 III [19]65 / Genitalia slide by RWP [male] USNM 45183 (1 male); Big Bend Nat[ional] Park, Grapevine Hill, 2 X [19]65, A. & M. E. Blanchard (1 male); Big Bend Nat[ional Park], Green Gulch, A. & M. E. Blanchard, 3 IV [19]65 (1 male); 1 VII [19]65 (1 male); 30 XI [19]65 / Barcode of Life, DNA voucher specimen CCDB-20821-C07, BOLD Proc. ID LNAUS 5029-13 / USNMENT 00906838 (1 male); Big Bend Nat[iona]l P[ar]k, Green Gulch, 30 XI [19]85, leg. E. C. Knudson / Barcode of Life DNA voucher specimen, SmpleID CCDB-20821-C07, BOLD Proc. ID LNAUS5028-13 / USNMENT 00906838 (1 male); Big Bend National Park, Oak Spring, 8 V 1959, M. R. MacKay (1 male); 4 X [19]65, A. & M. E. Blanchard (1 male); Terlingua Ranch, 29.43–[29].47° -103.38–[103].41°, 9 IV 2016, 1090–1135 m, LG Crabo/BC Schmidt (1 male); Jeff Davis County: Ft. Davis, 3 V 2000, J. Vargo / Database # CNCLEP 00113220 / Barcode of Life, DNA voucher specimen, SmplID CNCLEP 00113220, BOLD Proc. ID: CNCLB906-14 (1 male); W[est] Davis M[oun]t[ain]s/R[ou]te 166, 30.685° -104.244°, 11 IV 2016, 1888 m, L G Crabo/B C Schmidt (1 male); Terrell County: Sanderson, J. Vargo, 2 V 2000 / *Supralathosea* sp. det. Ed Knudson / Database # CNCLEP 00113933 / Barcode of Life Project Leg removed DNA extracted (1 male); 2 V 2000 / Database # CNCLEP 00114076 (1 female); Val Verde County: 29.805° -101.556°, 17 III 2018, J. Vargo leg. (1 male). CNC, JV, LGC, NMNH.

**Figures 26–29.**
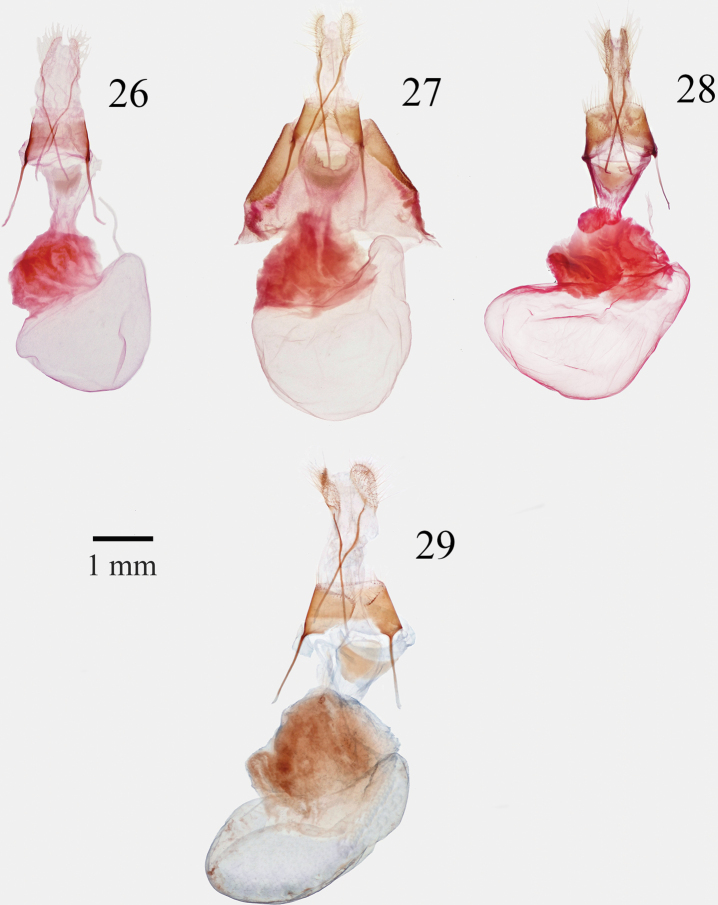
*Supralathosea* female genitalia **26***Supralathoseababoquivariensis***27***S.solastella* (Brewster County, Texas) **28***S.solastella* (Zapata County, Texas) **29***S.yavapai*.

**Figures 30–32.**
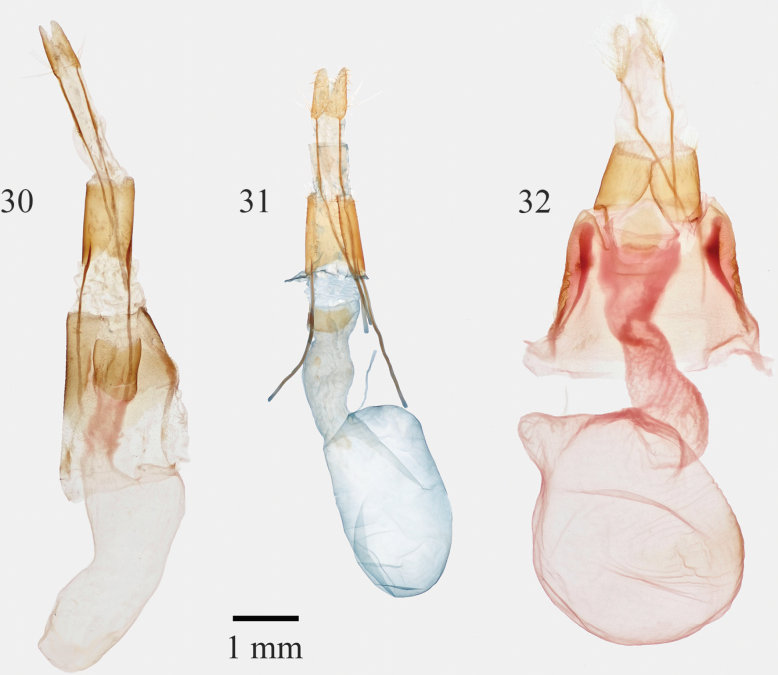
*Infralathosea* and *Eulathosea* female genitalia **30***Infralathoseapronuba***31***I.unicornis***32***Eulathoseaobtusa*.

**Figure 33.**
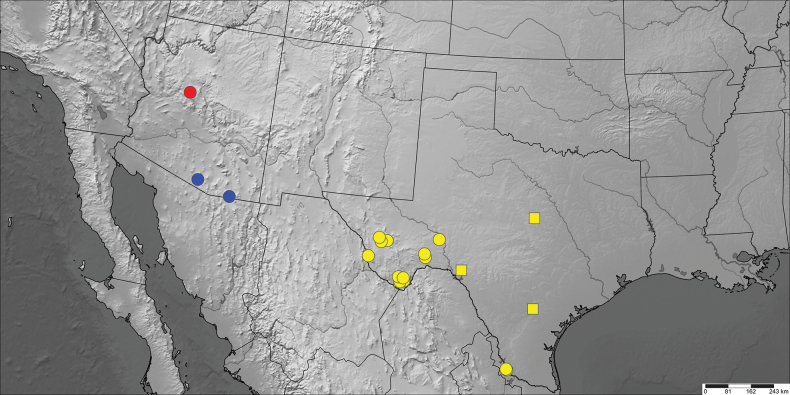
Distribution map of *Supralathosea* species. *Supralathoseababoquivariensis* (blue), *Supralathoseasolastella* (yellow), and *Supralathoseayavapai* (red). Circles = examined specimens. Squares = iNaturalist records.

**Figure 34.**
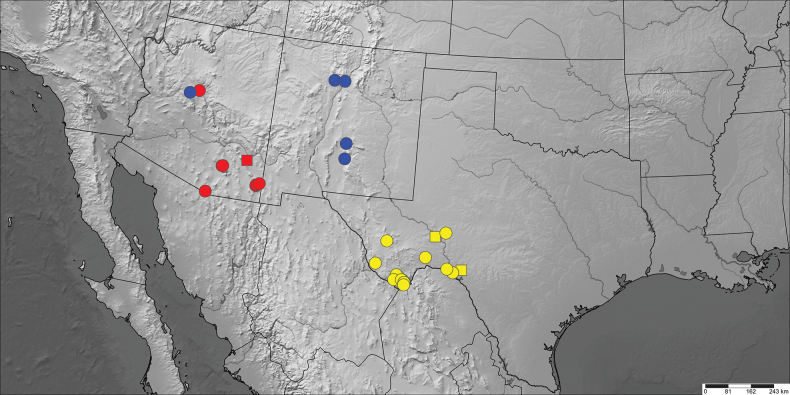
Distribution map of *Infralathosea* and *Eulathosea* species. *Infralathoseapronuba* (blue), *Infralathoseaunicornis* (yellow), and *Eulathoseaobtusa* (red). Circles = examined specimens. Squares = iNaturalist records.

The type series is restricted to west Texas.

##### Etymology.

The species name is Latin, meaning lone star. This species occurs in Texas, the Lone Star State.

##### Diagnosis.

This species is geographically variable with distinct phenotypes in west Texas and south Texas. All populations have paler gray forewings than those of *S.baboquivariensis*, and lack the rough texture of that species. Males of *S.solastella* from west Texas are dull medium gray with a black posterior margin; other markings are limited to slightly darker gray spots on the costa and terminal area patches. The populations from south Texas (Live Oak, Starr, and Zapata counties) are paler gray with a slight sheen, with distinct black transverse lines unlike males of any other *Supralathosea*. Like *S.baboquivariensis*, the male hindwing ground color of *S.solastella* is pure white, but dark scaling along the veins is less extensive in *S.solastella* than in *S.baboquivariensis*, with only a few dark scales on the veins at the positions of the postmedial and terminal lines. Females of *S.solastella* are darker and more mottled than the males, but the differences between the sexes are less pronounced than in *S.baboquivariensis*. As in the males, the forewing transverse lines are most prominent in the south Texas populations. Differences in size and habitus between *S.solastella* and *S.yavapai* are the same as those between *S.baboquivariensis* and *S.yavapai* as noted in the *S.baboquivariensis* Diagnosis section.

The male of *S.solastella* has a longer thinner pollex than that of *S.yavapai*. The valve and phallus of *S.solastella* are similar to those of *S.baboquivariensis* but are slightly more massive. These subtle differences are best appreciated when compared side-by-side. The female corpus bursae of *S.solastella* is larger than that of *S.baboquivariensis*, and has a longer rightward extension. It is slightly more globular than that of *S.yavapai*.

*Supralathoseasolastella* is the only known *Supralathosea* in Texas.

**Figure 35.**
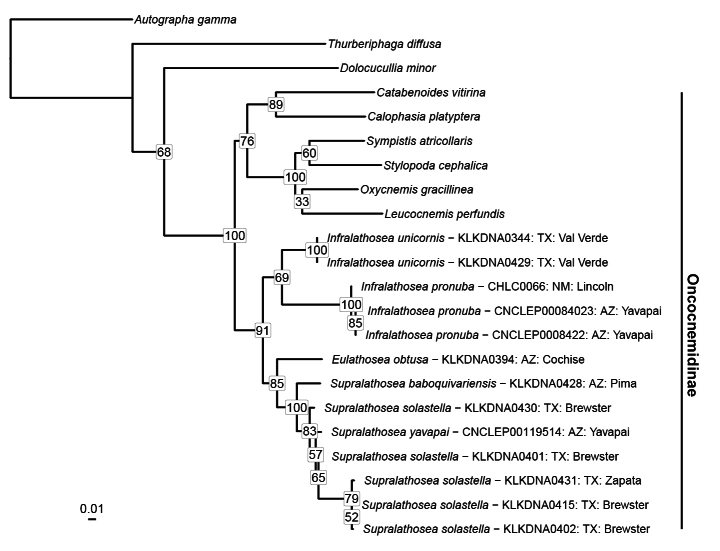
Maximum-likelihood tree inferred with RAxML based on five nuclear genes and CO1 of *Supralathosea*, *Infralathosea*, *Eulathosea*, selected Oncocnemidinae genera, and outgroups in other noctuid subfamilies (Plusiinae, Acontiinae, and Cuculliinae). Bootstrap values are shown over nodes. Tip labels for *Supralathosea*, *Infralathosea*, and *Eulathosea* provide additional identifying information in order to link specimens to full collection details.

The barcodes of *S.solastella* (*n* = 13) are assigned to four BINs in BOLD (BOLD:AAH5315, *n* = 9; BOLD:ADP4071, *n* = 1; BOLD:ADO7461, *n* = 1; BOLD:ADP4451; *n* = 2), and are not reliably differentiated from the single known haplotype of *S.yavapai* (*n* = 3). The two *S.solastella* specimens from the singleton BINs are from the same localities as some of the AAH5315 samples and the moths associated with these samples are not distinguishable by appearance, indicating that the genetic differences represent intraspecific variation. BOLD samples demonstrate minor barcode differences of approximately 1.4% between *S.solastella* populations from south Texas and those from west Texas (accessed May 2024); however, we did not recover each of these populations as monophyletic in our RAxML analysis (Fig. 35).

##### Description.

**Adults. *Head*** – Male antenna rami up to 2.5 × shaft width. Scape short, white, and dark gray. Labial palpus scales mostly flat dark gray and white, few hair-like. Frons white with black dorsal transverse bar; dorsum scales white-tipped gray and white. ***Thorax*** – Scales mostly white-tipped dark gray trifurcate, few gray hair-like, appearing powdery medium gray with black and pale gray transverse band on collar base and dark tuft on metathorax; venter gray. ***Legs***: Scales mixed mostly dark gray and scattered white; tarsal segments dark gray ringed with white. ***Wings***: Forewing length 12.0–15.5 mm (males); 14.5–16.5 mm (females), length 2.5 × width, scales, white, black-rimmed white, silvery gray, and black; male ground color uniform slightly powdery medium gray (west Texas) or uniform slightly glossy pale to medium gray (south Texas); posterior margin black, thickest in west Texas populations; three ill-defined gray patches in terminal area; veins slightly darker, powdery; antemedial and postmedial lines variable, often limited to gray marks on costa and a series of dark lines on the veins at the postmedial line (west Texas) or complete, black, sinuous, thickest anteriorly (south Texas); subterminal line faint, pale, strongest proximal to three ill-defined gray patches in terminal area; terminal line dark gray, strongest between veins; fringe weakly checkered ground color and dark gray; orbicular spot oval, faint, pale with punctate or linear ocellus; reniform spot absent or a pale smudge. Female forewing ground color slightly darker than that of males of the same population, less uniform with pale mottling near costa and on distal wing. Hindwing ground color pure white; males with scattered dark gray scales on distal veins and terminal line; females with variable powdery gray scales (most numerous in south Texas populations) forming diffuse discal spot, thin postmedial line and broad marginal band ending short of margin; fringe white. ***Abdomen*** – Vestiture mostly mixed gray and white flat scales with fewer white hair-like scales. ***Male genitalia***: Uncus and juxta as for genus. Valve shape as for genus, length 2.7 × width; sacculus 0.75 × valve length and 0.8 × valve width, dorsal margin broadly convex; ampulla length 0.5 × valve width; pollex spike-like, 0.5 × valve width. Phallus tubular, length 4 × width, apex as for genus. Vesica as for genus, slightly larger with longer stouter cornuti than that of *S.baboquivariensis*. ***Female genitalia***: Papilla analis shape and vestiture as for genus, length 2 × width. Segment A8 and apophyses as for genus. Ostium bursae and ductus bursae as for genus, rubbery bulb-like anterior ductus segment 2.1–2.3 × A8, about as wide as long, with perpendicular junction to corpus bursae. Corpus bursae shape as for genus, length 2.1–2.3 × A8, width 1.6–1.8 × length, globular, rightward conical projection 1.5 × A8.

##### Distribution and biology.

This species occurs in Texas. Examined specimens from west Texas are from the Chihuahuan Desert in the Big Bend region as far east as Terrell and Pecos counties. Examined specimens from south Texas are from Starr and Zapata counties. Many distribution records of *S.solastella* are from near the Rio Grande River indicating that this species undoubtedly occurs in adjacent Mexico, although its distribution south of the United States is unknown to us. Additional photographed live moths on iNaturalist (iNaturalist community 2024) likely representing this species from central Texas were examined from Bosque County (e.g., iNat/146854080), Hamilton County (e.g., iNat/146544200), and Val Verde County (e.g., iNat/104607005). These moths are most similar to west Texas populations of *S.solastella*, but their identification is hampered because the hindwings are not visible. In contrast, iNaturalist images from Live Oak County (e.g., iNat/214922172) are a close match to *S.solastella* from south Texas. The iNaturalist localities are included on the *S.solastella* distribution map (as squares) because they are the only known *Supralathosea* records from several parts of Texas (Fig. 35). *Supralathoseasolastella* flies during most of the year. The examined specimens were collected during March–April, June–early August, and late September–November. Most iNaturalist photographs of *Supralathosea* from Texas were taken during mid-winter, December–February. The larva feeds at night on new ash foliage (*Fraxinus* sp., Oleaceae) (David L. Wagner, unpub. data).

##### Discussion.

How to best classify the superficially distinctive *Supralathosea* populations from west and central Texas from those from south Texas was a difficult problem. We considered treating them as two species, subspecies of the same species, or—as presented—as distinctive unnamed populations of a single species. We hope that this paper will stimulate sampling of *Supralathosea* from regions between these populations, particularly from central Texas and northern Mexico, to corroborate or refute our thesis.

#### ﻿ Supralathosea yavapai sp. nov.

Taxon classificationAnimaliaLepidopteraNoctuidae

C416F346-317F-5F2A-B112-63C2BE0D75B3

https://zoobank.org/F8189AAC-49AF-478C-A472-58F5F63136FB

Figs 10–12 , 22 , 29 , 33 (Map)

##### Type material.

***Holotype*, male.** [USA]: ARIZ[ONA]: Yavapai Co[unty], 5 mi. [8.0 km] N[orth] [of] Prescott, 5450 ft [1661 m], 24 IV 1973, Lloyd M. Martin. CNC. ***Paratypes***, 83 males, 5 females. [**USA**]: **Arizona**: Yavapai County: Prescott, collected by Lloyd M. Martin: 31 I 1970 (1 male); 6 II 1970 (1 male); 7 II 1970 (1 male); 8 II 1970 (1 male); 9 II 1970 (1 male); 10 II 1970 (1 male); 11 II 1970 (1 male); 13 II 1970 (1 male); 17 II 1970 (1 male); 23 II 1970 (1 male); 24 II 1970 (1 male); 28 II 1970 (1 male); 8 III 1970 (1 female); 21 III 1970 (1 male); 23 III 1970 (1 male); 25 III 1970 (1 male); 7 IV 1970 / Genitalia slide by RWP [female] USNM 45182 (1 female); 8 IV 1970 / Genitalia slide by RWP [male] USNM 45181 (1 male); 9 IV 1970 (1 female); 10 IV 1970 (1 female); Prescott, collected by Lloyd M. Martin, 29 I 1970 / Granite Dells, 4 mi. [6.4 km] N[orth] of (1 male); 4 mi. [6.4 km] N[orth] of Prescott, Lloyd M. Martin: 16 II 1972 / Genitalia slide #NOC17486 / Specimen voucher #CNCLEP00140439 (1 female); 18 II 1972 (1 male); 21 II 1972 (9 males); 23 II 1972 (5 males); 25 II 1972 (2 males); 8 III 1972 (2 males); 9 III 1972 (1 male); 10 III 1972 (3 males); 17 III 1972 (3 males); 17 III 1972 / [Crabo genitalia slide] 658 male (1 male); 19 III 1972 (1 male); 20 III 1972 (2 males); 22 III 1972 (1 male); 5 mi. [8.0 km] N[orth] [of] Prescott, 5450 ft [1661 m], Lloyd M. Martin: 23 IV 1973 (2 males); 23 IV 1973 / CNC / Genitalia Slide by RWP, USNM 41,553 (1 male); 24 IV 1973 (5 males); 24 IV 1973 / Specimen ID CNCLEP 00119516 / Barcodes of Life Project Leg removed DNA extracted (1 male); 24 IV 1973 / Specimen ID CNCLEP 00119514 / Barcodes of Life Project Leg removed DNA extracted (1 male); 24 IV 1973 / Genitalia CNC slide # 13942 male (1 male); 25 IV 1973 (5 males); 25 IV 1973 / Genitalia CNC slide # 13548 male (1 male); 26 IV 1973 (2 males); 27 IV 1973 (7 males); 27 IV 1973 / Genitalia CNC slide # 17796 male (1 male); 28 IV 1973 (3 males); 28 IV 1973 / CNC / Genitalia Slide by RWP, USNM 41,569 (1 male); 5 V 1973 (3 males); 5 V 1973 / Specimen ID CNCLEP 00119515 / Barcodes of Life Project, Leg removed, DNA extracted (1 male). CMNH, CNC, LACM, LGC, NMNH.

##### Etymology.

The species epithet is the name of the people who inhabited Arizona between the Gila and Colorado rivers for hundreds of years prior to the colonization of the West by Caucasians of European descent. All examined specimens of this species are from Yavapai County, Arizona.

##### Diagnosis.

*Supralathoseayavapai* males are distinguished by their off-white hindwing ground color, pure white in other *Supralathosea* species. The male antenna is the widest in the genus, rami 3.0 × shaft width (2.5 × in the other species). It is the largest *Supralathosea*, forewing length at least 16.5 mm (< 15.5 mm in the other species).

Males of *S.yavapai* have a relatively short broad-based triangular pollex. Those of other *Supralathosea* species are longer and thinner. The female genitalia of *S.yavapai* are only subtly different from those of other *Supralathosea* species. The crenulate anterior segment of the ductus bursae is relatively large compared to the bursa copulatrix in *S.yavapai*, and the membranous bursa is wider than long in *S.yavapai*, longer than wide in the other *Supralathosea* species.

The barcode of *S.yavapai* (BOLD:ADP3298; *n* = 3) is similar to several barcode haplotypes of *S.solastella*, clustering with them on similarity trees. These species are not recovered as distinct in our RAxML analysis (Fig. 35).

##### Description.

**Adults. *Head*** – Male antennal rami up to 3 × central shaft width. Scape pale gray, tuft long, loose. Labial palpus as for genus, flat scales mixed dark gray and white. Frons scales gray, with a loose median ridge; dorsum scales long, pale gray, white-tipped gray, and black, darkest centrally; loose tufts anterior and posterior to antenna. ***Thorax*** – Scales long, white-tipped gray and scattered black or white, long hair-like black scattered on tegula and dense on dorsum, appearing medium gray (male) or charcoal gray (female) with black and pale gray bands across collar base and loose dark tuft on metathorax; venter densely hairy, dark gray. ***Legs***: As for genus. ***Wings***: Forewing length 16.5–18.5 mm (males); 17.5–18.0 mm (females), length 2 × width; scales white, black-rimmed white, and medium to dark gray; male ground color uniform slightly powdery medium gray with slight brownish tint, terminal area slightly darker, trailing margin blackish gray; veins slightly darker, powdery; basal line absent; antemedial line an oblique gray smudge on costa, occasionally dark marks on veins; medial line a gray smudge on costa or absent; postmedial line small dark marks on costa and series of dark lines on M3 to 1A+2A, strongly oblique basad posterior to cell; subterminal line absent or ill-defined, pale gray, with or without faint proximal darker shade; faint darker patches in terminal area; terminal line dark gray, strongest between veins; fringe weakly checkered ground color and slightly darker gray; orbicular and reniform spots absent or very faint; female ground color powdery charcoal gray, darkest centrally; with indistinct intervenal dark gray in terminal area; antemedial, medial, postmedial, and subterminal lines present, stronger than in males; orbicular and reniform spots evident as pale filling against dark ground color. Hindwing pale yellowish gray, gray suffusion along anterior margin (male) or diffuse (female); veins powdery gray; discal spot small, gray; terminal line powdery gray, thicker between veins; fringe white (male), white with incomplete gray base (female). ***Abdomen*** – Scales flat and hair-like, gray. ***Male genitalia***: Uncus as for genus, mesial and subapical segments widest. Juxta ventral extension long. Valve shape as for genus, length 3.6 × width, sacculus 0.75 × valve length, dorsal margin broadly convex; clasper as for genus; pollex relatively short, triangular with broad base, length 0.25 × valve width. Phallus length 4 × width. Vesica relatively large, similar to that of *S.solastella* but slightly more massive. ***Female genitalia***: Papilla analis as for genus, length 2 × width. A8 length 0.5 × width. Posterior apophysis 2.3 × A8; anterior apophysis 0.6 × posterior apophysis. Ostium bursae and ductus bursae as for genus, anterior segment as long as wide, 2 × A8, broad junction to corpus bursae angled 30°. Corpus bursae as for genus, width greater than length, with relatively long rightward extension.

##### Distribution and biology.

This species is known from nearly 100 specimens collected by Lloyd Martin at his home north of Prescott, Arizona in the early 1970s, most of which are now at CNC and LACM. The habitat is dry ponderosa pine forest (J. D. Lafontaine pers. comm. 2018). All specimens were collected from late January to early May. This species might be more widespread in the Southwest United States but might elude detection due to its early flight period. The early stages of *S.yavapai* are unknown.

##### Discussion.

The dense vestiture of the moth is probably an adaptation to cold temperatures during its spring flight.

#### ﻿Genus Infralathosea gen. nov.

Taxon classificationAnimaliaLepidopteraNoctuidae

BD68776A-A84A-5D57-A174-FDF01E026A0A

https://zoobank.org/7DD5A023-E8DE-4CAE-A3BE-EBDBDA5C0D60

##### Type species.

*Catabenapronuba* Barnes & McDunnough, 1916.

##### Gender.

Feminine.

##### Etymology.

The name is derived from the Latin prefix *infra* meaning below, and *Lathosea*, the genus included in the name *Supralathosea*. *Lathosea* is a junior subjective synonym of *Cucullia* Schrank (Noctuidae, Cuculliinae).

##### Diagnosis.

*Infralathosea* adults are relatively small noctuid moths, forewing lengths 12.0–14.5 mm, with streaky pale gray forewings. The antemedial and postmedial lines are fused in the anterior and posterior medial area, forming thin black arcs based on the costa and posterior margin. The anterior arc is strongest and is diagnostic. Other markings include a thin basal dash, subterminal intervenal black lines, and indistinct darker gray patches on the costa (especially at the medial line origin), apex, and trailing margin. The orbicular and reniform spots are reduced to small pale patches. The hindwings are nearly pure white, except gray in the female of one species. The antennae of both sexes are thin, beaded in males.

*Infralathosea* species resemble *Catabena* Walker and *Catabenoides* Poole species (both Noctuidae, Oncocnemidinae). *Infralathosea* species have the forewing lines fused into arcs; lines absent or typically noctuiform in *Catabena* and *Catabenoides*, extending from the costa to the posterior margin when visible.

Males of *Infralathosea* have a cylindrical arced uncus, lacking a focal bend like those of *Supralathosea* and *Eulathosea*. The valves are simple, strap-like with a weak sacculus, thin curved thorn-like ampulla, and bluntly pointed apex lacking an expanded cucullus, pollex, or corona. Patches of short cornuti on the tubular vesica are arranged in patches on subbasal and medial bulges, with a stout subapical cornutus in one species. Females have a long conical papilla analis. The distal abdominal intersegmental membranes and apophyses are long and the ovipositors protrude from the distal abdomen in situ. The ductus bursae is tubular, membranous except for sclerotized postvaginal plates, and the corpus bursae is elongate, slightly asymmetric with the ductus seminalis joined to the posterior end. *Infralathosea* females differ from *Supralathosea* and *Eulathosea* females in many respects, including the elongate posterior abdominal structures; those of both other genera have nonprotruding pad-like papillae, short intersegmental membranes, and short apophyses.

##### Description.

**Adults. *Head*** – Male antenna bead-like, setose ventrally; female antenna filiform, setae sparse. Scape medium length. Labial palpus as in *Supralathosea*, white and gray. Frons convex, scales white with dorsal transverse black line. Head scales white, gray, and white-tipped gray. Haustellum present. ***Thorax*** – Scales dimorphic, long narrow flattened or hair-like; white, gray, or white-tipped gray; forming slight hoary medium gray crest; venter pale gray. ***Wings***: Forewing length 12.0–14.5 mm (males), 13.5–14.5 mm (females), apex blunt; scales strap-like white, gray, and black, appearing slightly streaky pale gray; lines black, thin, indistinct except on costa, anterior segments of antemedial and postmedial lines fused posterior to Cu and posterior segments fused near posterior margin, forming anterior and posterior arcs; orbicular spot small pale with central dark line; reniform spot a pale smudge or absent; claviform spot absent. Male hindwing ground color pure white; thin band of off-white scales along dorsal anterior margin towards apex; Female hindwing ground color either white with scattered gray scales near margin and on veins in some individuals (1 species) or medium gray (1 species). ***Legs***: Lacking spines or claws; scales dark gray and white; tarsi dark gray with distal white rings; ventral tarsal segments except distal segment with three regular rows of uniform spiniform setae. ***Abdomen*** – Lacking dorsal A1 transverse channel, brush organs, and coremata. Scales mostly flat, fewer hair-like, medium gray. ***Male genitalia***: Uncus thin, arced. Juxta shield-shaped, ventral margin broadly V-shaped. Valve simple, strap-like, length 3.5 × width; apex pointed bluntly, lacking corona or pollex; sacculus length 0.4 × valve length, width 0.5 × valve width, dorsum straight or weakly convex; clasper base a sclerotized rod at ventral margin distal to sacculus, ampulla thorn-like, base directed 45° dorsad from origin at valve distal 1/3, distal segment directed dorsad and slightly mesially beyond 90° bend. Phallus tubular, length 4 × width. Vesica length 1 × phallus length, diameter 0.4–0.5 × length, nearly straight beyond basal 90° bend; proximal vesica with broad-based dorsal bulge; bulge and adjacent posterior vesica surface covered by multiple porrect spine-like cornuti, much less massive than those of *Supralathosea*; mid vesica with broad posterior bulge or diverticulum covered by loose cluster of shorter thinner cornuti; single stout apical cornutus (1 species). ***Female genitalia***: Segment A7 unmodified. Segment A8 elongate, width 0.6–0.7 × length, sparse short setae densest at posterior margin. Papilla analis 0.6–0.7 × A8, width 0.3–0.4 × length, mediolaterally compressed, apex pointed bluntly; sparse long hair-like setae at base and multiple short thin setae on distal third; membrane to A8 long, 1.8 × A8. Posterior apophysis 2.5–3.0 × A8; anterior apophysis 0.65–0.7 × posterior apophysis. Ostium bursae shallow, quadrate, membranous. Ductus bursae tubular, slightly expanded mesially in 1 species, length 2 × A8, weakly sclerotized except for strong quadrate ventral and dorsal postvaginal plates on posterior 1/5 to 1/3, joined broadly to left posterolateral corpus bursae. Corpus bursae membranous, lacking signa, length 2.3–2.6 × A8 and width 0.33–0.5 × length, elongate, ovoid, ductus seminalis at medial posterior end.

##### Higher classification.

*Infralathosea* is assigned to Oncocnemidinae (Keegan et al. 2021). The male genitalia, including the simple valve with thorn-like ampulla and tubular vesica with patches of cornuti, are typical of the subfamily.

**Distribution and biology**: Like *Supralathosea*, this genus has a limited distribution in the American Southwest, occurring in central Arizona, New Mexico, and west Texas. At least one of the species probably occurs in adjacent northern Mexico. Adults of *Infralathosea* fly during much of the year except in the winter, with most records from spring and fall. The early stages are unknown. The papilla analis shape and long eversible ovipositor suggest that the eggs might be inserted into its hostplant, and as such the larva may be an internal feeder for at least a portion of its larval stage.

##### Discussion.

This genus includes two allopatric species, *Infralathoseapronuba* comb. nov. from central Arizona and New Mexico and *Infralathoseaunicornis* sp. nov. from west Texas. The males are indistinguishable without dissection or barcoding, while females are best distinguished by superficial appearance. As discussed above under *Supralathosea*, *Infralathosea* species lack an A1 transverse channel. *Catabenapronuba* was associated with *Supralathosea* by Troubridge (2008) based on the absence of this character.

#### ﻿ Infralathosea pronuba

Taxon classificationAnimaliaLepidopteraNoctuidae

(Barnes & McDunnough, 1916) comb. nov.

0E3CFE64-5C03-5E2F-A550-0BA368BE1CAD

Figs 13 , 14 , 23 , 30 , 34 (Map)

Catabena pronuba Barnes & McDunnough, 1916: 10, pl. 3 fig. 11.
Cucullia nanuscula Dyar, 1922: 168.

##### Type material.

*Catabenapronuba*: Holotype, male, USA, New Mexico, Jemez Springs. NMNH. [examined]. *Cuculliananuscula*: Holotype, male, USA, New Mexico, Jemez Springs. NMNH. [examined].

##### Diagnosis.

*Infralathoseapronuba* males are superficially identical to *I.unicornis*. Both are relatively small streaky gray moths with characteristic fused forewing lines as described in the genus description and have nearly pure white hindwings. They are distinguishable by their genitalia. Males of *I.pronuba* lack an apical cornutus on the vesica, present in *I.unicornis*.

The females of *Infralathosea* species have identical forewings and similar genitalia, but are distinguished easily by their hindwings, gray in *I.pronuba* and white in *I.unicornis*.

*Infralathoseapronuba* is the only *Infralathosea* species in Arizona and New Mexico. *Infralathoseaunicornis* occurs in west Texas.

The barcode of *I.pronuba* (BOLD:ACM7023, *n* = 13) differs by 5.3% from that of *I.unicornis*.

##### Distribution and biology.

This species is known from north and central New Mexico and central Arizona (Yavapai County).

*Infralathoseapronuba* flies during spring and summer. Most examined specimens were collected from early March through April, with additional records from July. This species is not rare in central New Mexico, but there are relatively few specimens in examined collections. The early stages are unknown.

#### ﻿ Infralathosea unicornis sp. nov.

Taxon classificationAnimaliaLepidopteraNoctuidae

6B2CC2E0-69EA-5EDD-AD02-59806016945F

https://zoobank.org/AC2731C6-182D-4963-B206-42764A7BDE95

Figs 15 , 16 , 24 , 31 , 34 (Map)

##### Type material.

***Holotype*, female.** [**USA**]: **Texas**: [Brewster County]: Big Bend N[ational] P[ark], Chihuahuan Desert near Nugent M[oun]t[ai]n, 1 X [19]67, A. & M. E. Blanchard. NMNH. ***Paratypes***, 112 males, 49 females. **USA: Texas**: Brewster County: Big Bend N[ational] P[ark], Chihuahuan Desert near Nugent M[oun]t[ai]n., A. & M. E. Blanchard, 1 X [19]67 (2 males); 8 X [19]69 (1 female); Big Bend Nat[ional] Park, Dugout Wells, A. & M. E. Blanchard, 28 IX [19]65 (3 males, 5 females); 28 IX [19]65 / Genitalia slide by RWP [female] USNM 45299 (1 female); 3 X [19]66 (1 female); 30 III [19]71 (1 male); Big Bend National Park, Glenn Springs R[oa]d, near Nugent M[oun]t[ai]n., Leg. E. C. Knudson, 28 IX [19]81 (1 male); Big Bend Nat[ional] Park, Gov[ernment] Spring, A. & M. E. Blanchard, 29 IX [19]65 (7 males, 4 females); 29 IX [19]65 / [male] genitalia on slide A. B. 202 (1 male); 29 IX [19]65 / Genitalia slide by R. F. USNM 42,598 (1 female); 4 X [19]67 (1 male); 27 III [19]71 (2 males); Big Bend Nat[ional] Park, near Nugent M[oun]t[ai]n, leg. E. C. Knudson, 13 IX [19]82 (1 male); Big Bend Nat[ional] Park, Oak Spring, A. & M. E. Blanchard, 2 IV [19]65 (1 male); 6 X [19]65 (1 female); Big Bend Nat[ional] Park, K-Bar Res[earch] Sta[tion], A. & M. E. Blanchard, 23 III [19]71 (2 males, 1 female); 23 IX [19]71 (1 female); 25 IX [19]71 (1 female); Terlingua Ranch, 29.43–[29].47° -103.38–[103].41°, 1090–1135 m, 9 IV 2016, L. G. Crabo/B. C. Schmidt / Genitalia slide # 17800 [male] (1 male); Terlingua Ranch, guest cabin, 29.452° -103.393°, 1135 m, 9 IV 2016, UV lt., C. Schmidt, J. Dombroskie, L. Crabo CNC535745 (1 male); Terlingua Ranch, Church Rd., 29.442° -103.386°, 1092 m, 9 IV 2016, C. Schmidt/J. Dombroskie/L. Crabo / Database # CNCLEP 535915 / Barcodes of Life Project, Leg removed, DNA extracted (1 male); Terlingua Ranch Lodge, 29.453° -103.388°, 18 IX 2019, Limestone outcrop, C. Jaeger / CNCLEP numbers: 00232026–00232028, 00272842–0027845, 00272847, 00272848, 00272850–0027853, 00272885, 00272894, 00272895, 00272906, 00272907, 00272909, 00272912, 00272914, 00272915, 00272920, 00272929, 00272930, 00272958, 00272982, 00272988, 00272990, 00273064–00273067, 00273069, 00273070, 00273072–00273075, 00273742, 00273744–00273746, 00273784–0273786 (46 males); numbers: 00232025, 00272908, 00272910, 00272911, 00272927, 00272976, 00272980, 00272986, 00273062, 00273063, 00273068, 00273071, 273723, 00273726, 00273747, 00273748, 00273771, 00273787, 00272846, 00272849, 00272854, 00272855, 00272856 (23 females); Terlingua Ranch Lodge, Whitehouse Mtn saddle, 29.453° -103.388°, 22 IX 2023, Limestone outcrop, C. Schmidt, C. Jaeger / CNCLEP numbers 00335508, 00335510, 00335511, 00335513, 00335520, 00335537, 00335561, 00335562, 00335581, 00335582, 00335594, 00335620–003356213, 00335681, 00335685, 00335689, 00335719, 00335733, 00335734, 00335842 (22 males); Presidio County: Shafter, 9 IX [19]69, A. & M. E. Blanchard (4 males, 1 female); Terrell County: Sanderson, Desert Air Motel, N30.1453° W102.4059°, elev. 2810’ [856 m], 27 + 28 IX 2024, to MV/BL, Leg. D. L. Wikle (11 males, 4 females); Val Verde County: Langtry, N29.804° W101.557°, 7 III 2016, J. Vargo (1 male); Same collection label / Crabo slide 629 [female] (1 female); Same locality, collector 17 III 2018 (1 male, 3 females); Seminole Canyon, 1400 ft. [427 m], N29.696° W101.324°, 22 III 2016, J. Vargo (2 males). CNC, DLW, JV, LGC, NMNH.

##### Etymology.

The name is Latin, meaning one horn. It refers to the diagnostic stout cornutus on the distal male vesica.

##### Diagnosis.

This species resembles *I.pronuba* in habitus and structure. Males *I.unicornis* are superficially identical to those of *I.pronuba*, but can be identified by a stout cornutus on the distal vesica that *I.pronuba* lacks. The females of these species also have identical forewings, but the hindwing ground color is nearly pure white in *I.unicornis*, gray in *I.pronuba*.

*Infralathoseaunicornis* is the only species in the genus in west Texas. The barcode of *I.unicornis* (BOLD:ADF0036; *n* = 1) differs from that of *I.pronuba* by 5.3%.

##### Description.

**Adults. *Head*** – Antennae of both sexes as for genus. Scape tuft medium dark gray. Labial palpus scales dark gray. Frons white, transverse black dorsally; dorsal head scales white and white-tipped gray, loose paramedian tufts anterior and posterior to antenna. ***Thorax*** – Patagium scales spatulate, white-tipped, appearing gray with weak dark basal line. Tegula scales long, spatulate, white, with fewer black of similar shape and hair-like, appearing pale gray; dorsal thorax charcoal; venter pale gray. ***Wings***: Forewing length 13.0–14.0 mm; scales white, pale gray, dark gray; appearing slightly streaked medium gray, darkest on mesial posterior margin and in terminal area near apex and anal angle; veins and intervenal dark lines near apex dark gray; basal dash black; antemedial and postmedial lines fused as in genus description, anterior antemedial line and mesial fused segment darkest; medial line a gray smudge near costa; subterminal line diffuse, irregular, pale gray; fringe white and gray, slightly darker between veins; spots as for genus. Hindwing ground color pure white (male); uniform medium gray (female). ***Legs***: As in genus description. ***Abdomen*** – Vestiture as for genus. ***Male genitalia***: Uncus and juxta as for genus. Valve, including sacculus, clasper, and cucullus as for genus. Phallus length 4 × width. Vesica length 1 × phallus, width, diameter 0.4 × length; proximal cornuti patches as in genus description; mesial cornuti on distinct dome-shaped diverticulum; posterior apex with single stout cornutus. ***Female genitalia***: Papilla analis, intersegmental membrane, and A8 as for genus. Posterior apophysis 3.0 × A8; anterior apophysis 0.65 × posterior apophysis. Ostium bursae as for genus; postvaginal plate length 0.2 × ductus bursae; ductus bursae 1.9 × A8, widest mesially. Corpus bursae shape as for genus, length 2.3 × A8 and width 0.5 × length.

##### Distribution and biology.

This species occurs in Chihuahuan shrub desert in west Texas and is relatively common in collections. Most examined specimens are from the Big Bend region between Fort Davis and Big Bend National Park. Collection records suggest the presence of two broods, with records from spring (February to April) and fall (September and October). The early stages are unknown.

#### ﻿Genus Eulathosea gen. nov.

Taxon classificationAnimaliaLepidopteraNoctuidae

9448D76E-67C6-5CA1-9900-7456F13065FD

https://zoobank.org/D3BB84B1-B611-4BB6-8996-28F580FCF55A

##### Type species.

*Cuculliaobtusa* Smith, 1909.

##### Gender.

Feminine.

##### Etymology.

The name is derived from the Greek prefix *eu*, meaning good or pleasant, and *Lathosea*, the noctuid genus that is part of the names *Supralathosea* and *Infralathosea*. It refers to the attractive appearance of the only known species.

##### Diagnosis.

*Eulathosea* is a monotypic genus which differs in many ways from *Supralathosea* and *Infralathosea*. Adults (forewing length 17.0–18.5 mm) are larger than all species in the other genera other than *S.yavapai*. They can be identified without dissection by the solid dark hindwing marginal band, marginal band absent or at most a powdery mixture of gray and whitish scales in *Supralathosea* and *Infralathosea*, and streaky pale gray forewing with orange-tan scales in the cell that extend nearly to the distal margin, forewing gray without warm colors in the other genera.

The male uncus of *Eulathosea* is bent ventrad at a sideways-D-shaped thickening, beyond which it is gracile and arced. The valve is rhomboid, broadest at the midpoint with a V-shaped bulge at the distal sacculus, gradually tapering to a weak cucullus with apical and anal points and partial corona of claw-like setae on the dorsal two-thirds, and a long acute ampulla that reaches the dorsal margin. The vesica is bent 270°, with dilated proximal and distal segments bearing cornuti shorter than those of *Supralathosea* and longer than those of *Infralathosea*. Females have a rounded corpus bursae with thick rippled membrane and a cone-shaped leftward process bearing the ductus seminalis. The undulating tubular ductus bursae is moderately thick and rubbery, but lacks the bulbous anterior segment that is present in *Supralathosea* females.

##### Description.

**Adults.** Males and females superficially similar. ***Head*** – Antenna of both sexes filiform. Scape medium length, gray. Eyes lacking interommatidial setae, with few adjacent dark lash-like scales. Labial palpus distal segment length 0.4 × second segment, directed anterodorsad. Frons slightly convex, scales gray with black dorsal transverse line; dorsal head scales pale to dark gray, weak paramedian tufts gray anteriad and black posteriad to antenna. ***Thorax*** – Scales mostly long white-tipped pale gray, appearing pale gray; collar with basal tan, black, and pale gray lines, crest prominent in resting moths; weak tufts on metathorax; venter scales hair-like, white and pale gray. ***Legs***: Tibia lacking spines or claws; tarsal segments with three regular rows of spine-like setae. ***Wings***: Forewing length 17.0–18.5 mm, length 2.0–2.2 × width, outer margin smooth; scales strap-like white, pale to dark gray, tan; veins dark gray; appearing streaked and mottled medium gray, with dark patches on costa, distal posterior margin, and subterminal area, and pale orange-tan from cell base to wing apex; basal line absent; antemedial and medial lines dark oblique spots on costa; postmedial line with dark gray medial and whitish outer components, faint, irregular, drawn strongly basad in fold; subterminal line irregular, whitish, strongest between veins opposite cell and in fold; terminal line thin, dark gray; orbicular and reniform spots pale, small, faint; claviform spot absent; fringe weakly checkered ground color and dark gray. Hindwing ground color pearlescent white, veins and broad marginal band dark gray; fringe white, base striped ochre and dark gray. ***Abdomen*** – Lacking dorsal A1 transverse channel, brush organs, and coremata; scales gray; small dorsal tufts on segments A2 and A3. ***Male genitalia***: Uncus base stout, cylindrical, directed posteriad, bent 90° ventrad at sideways-D-shaped subbasal bulge, mid and distal segments gracile, arced, tip with small downward tooth. Juxta V-shaped, dorsal arms curved mesially. Valve turkey-drumstick-shaped with strongly convex ventral margin to mid valve and undulating weakly convex dorsal margin, distal valve tapered to weak rhomboid cucullus with blunt triangular apex and acute granulose anal angle lacking a pollex, with corona of 10–15 long claw-like setae dorsal 2/3; sacculus 0.5 × valve length, proximal 2/3 uniform, 0.3 × valve width, distal 1/3 dorsally convex with thick sideways-V-shaped bulge; clasper base a thick bar at distal sacculus, clasper ampulla directed distal and 45° dorsad from origin on ventral third of valve distal to sacculus, slightly curved, slender, thorn-like, reaching valve dorsal margin. Phallus tubular, length 4 × width, spinulose apex bulging slightly leftward with ventral and dorsal extensions onto vesica. Vesica bent 90° ventrad at base, then curved dorsad 180° with apex to right of mid-phallus; subbasal and distal vesica dilated with intervening waist, subbasal bulge 1.8 × phallus diameter, distal bulge 2.5 × phallus diameter with smooth taper to apex; multiple similar needle-like cornuti in ovoid patch on left side of subbasal bulge and T-shaped patch on left and ventral sides of distal bulge. ***Female genitalia***: A7 tergite normal size, pleural membrane thick, with anterior dark internally concave rugose structure (possibly a gland). Segment A8 width 1.5 × length. Papilla analis 0.8 × A8, width 0.3–0.4 × length, apex blunt, covered by uniform hair-like setae. Posterior apophysis 2.5 × A8; anterior apophysis 0.6 × posterior apophysis. Ostium bursae quadrate, width 0.5 × A8, ventral margin thin, sclerotized, postvaginal plates small; ductus bursae thick, rubbery, externally rugose, length 3 × A8, undulating, narrowest at midpoint, widest proximal to 90° ventrad bend at junction with corpus bursae. Corpus bursae globose, length and width 3 × A8, with short conical left ventral protrusion with ductus seminalis at apex and slightly thickened concentrically rippled membrane on right but lacking smaller distinct signum.

##### Higher classification.

*Eulathosea* is assigned to Oncocnemidinae (Keegan et al. 2021).

##### Discussion.

*Eulathosea* lacks an A1 transverse channel, as discussed under *Supralathosea*.

#### ﻿ Eulathosea obtusa

Taxon classificationAnimaliaLepidopteraNoctuidae

(Smith, 1909) comb. nov.

86E7D63F-9792-59D3-842C-9A024469CD66

Figs 17 , 18 , 25 , 32 , 34 (Map)

Cucullia obtusa Smith, 1909: 63.

##### Type material.

***Lectotype*, male**, designated by Todd 1982: 157. USA Arizona, Santa Catalina Mts. AMNH [examined from photograph].

##### Diagnosis.

*Eulathoseaobtusa* can be identified without dissection by the combination of streaky forewing pattern with orange-tan scales in and distal to the cell and white ground color hindwing with broad solid blackish marginal band that extends uninterrupted to the wing margin. It is most likely to be confused with streaky species of *Sympistis* Hübner (Noctuidae, Oncocnemidinae) or a *Cucullia* Shrank (Noctuidae, Cuculliinae) species with reddish brown on the anterior forewing (such as *Cuculliaeucaena* Dyar). Most *Sympistis* species have a claw-like seta on the distal foretibia that *Eulathosea* lacks. Species of these genera lack the combination of genitalia characters described in the *Eulathosea* Diagnosis section, above.

The barcode BIN of *E.obtusa* is BOLD:AAH9892.

##### Distribution and biology.

*Eulathoseaobtusa* occurs in forests of eastern and central Arizona. It flies during late summer and fall, with records of examined specimens between late July and early October. The early stages are unknown.

## Supplementary Material

XML Treatment for Supralathosea

XML Treatment for Supralathosea baboquivariensis

XML Treatment for Supralathosea solastella

XML Treatment for Supralathosea yavapai

XML Treatment for Infralathosea

XML Treatment for Infralathosea pronuba

XML Treatment for Infralathosea unicornis

XML Treatment for Eulathosea

XML Treatment for Eulathosea obtusa

## ﻿Additional information

### Author ORCIDs

Lars G. Crabo https://orcid.org/0009-0004-1089-5511

Kevin Keegan https://orcid.org/0000-0002-0872-7875

### Data availability

All of the data that support the findings of this study are available in the main text.
